# Superconducting spin valve effect in Co/Pb/Co heterostructures with insulating interlayers

**DOI:** 10.3762/bjnano.15.41

**Published:** 2024-04-25

**Authors:** Andrey A Kamashev, Nadir N Garif’yanov, Aidar A Validov, Vladislav Kataev, Alexander S Osin, Yakov V Fominov, Ilgiz A Garifullin

**Affiliations:** 1 Zavoisky Physical-Technical Institute, FRC Kazan Scientific Center of RAS, 420029 Kazan, Russia; 2 Leibniz Institute for Solid State and Materials Research, Helmholtzstr. 20, D-01069 Dresden, Germany; 3 L. D. Landau Institute for Theoretical Physics RAS, 142432 Chernogolovka, Russia; 4 Laboratory for Condensed Matter Physics, HSE University, 101000 Moscow, Russia

**Keywords:** ferromagnet, insulator layers, proximity effect, superconducting spin-valve, superconductor

## Abstract

We report the superconducting properties of Co/Pb/Co heterostructures with thin insulating interlayers. The main specific feature of these structures is the intentional oxidation of both superconductor/ferromagnet (S/F) interfaces. We study the variation of the critical temperature of our systems due to switching between parallel and antiparallel configurations of the magnetizations of the two magnetic layers. Common knowledge suggests that this spin valve effect, which is due to the S/F proximity effect, is most pronounced in the case of perfect metallic contacts at the interfaces. Nevertheless, in our structures with intentionally deteriorated interfaces, we observed a significant full spin valve effect. A shift of the superconducting transition temperature *T*_c_ by switching the mutual orientation of the magnetizations of the two ferromagnetic Co layers from antiparallel to parallel amounted to Δ*T*_c_ = 0.2 K at the optimal thickness of the superconducting Pb layer. Our findings verify the so far unconfirmed earlier results by Deutscher and Meunier on an F1/S/F2 heterostructure with oxidized interlayers [Deutscher, G.; Meunier, F. *Phys. Rev. Lett.* **1969**, *22*, 395. https://doi.org/10.1103/PhysRevLett.22.395] and suggest an alternative route to optimize the performance of superconducting spin valves.

## Introduction

Models and specific realizations of the superconducting spin valve (SSV) have been the subject of intensive research over the past 25 years [1–10]. The interest in these structures is due to the possibility to observe and exploit the reciprocal influence of superconductivity (S) and ferromagnetism (F) on each other when they are put into a close contact [11–17]. Moreover, SSV structures appear as promising devices for applications in modern superconducting spintronics [18–22]. In 1997, Beasley and coworkers proposed a theoretical F1/F2/S model of the SSV structure [1]. Another F1/S/F2 model was developed a little later in 1999 by Tagirov [2] and Buzdin and coworkers [3]. In these structures, F1 and F2 are metallic ferromagnetic layers, and S is a superconducting layer. Both models analyze the penetration of Cooper pairs from the S layer into the F layers under the action of the exchange field generated by the F1 and F2 layers. Those early theoretical works implied the operation principle of the SSV structure based on the control of the average exchange field acting on the S layer by changing the mutual orientation of the magnetization vectors of the F layers and, thus, suppressing superconductivity to a different degree. Typically, the superconducting transition temperature *T*_c_ of the SSV is minimal/maximal for the parallel (P)/antiparallel (AP) geometry of the two vectors, respectively. The magnitude of the SSV effect is defined as the difference of these two temperatures 
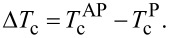
 The full SSV effect is realized when Δ*T*_c_ is larger than the superconducting transition width δ*T*_c_ in the P and AP configurations. Several experimental works confirmed the predicted influence of the mutual orientation of the magnetization vectors of the F layers on *T*_c_ in the F1/S/F2 type of structures [4–6,23–25]. However, a full switching between the normal and the superconducting state was not achieved because in these SSVs Δ*T*_c_ was always smaller than δ*T*_c_. For the first time, a complete switching between the normal and superconducting states was observed in the F1/F2/S type SSVs in [10].

Theories [13,15–16,26–28] predict that under certain conditions, a long-range triplet component (LRTC) in the superconducting condensate can arise in the S/F bilayer. The generation of the LRTC opens an additional channel for the leakage of the Cooper pairs from the S into the F layers in the F1/F2/S SSV at noncollinear configuration of F1 and F2 magnetizations. This suppresses *T*_c_ significantly and, thus, should manifest as a minimum of *T*_c_ at the orthogonal magnetizations’ geometry [29]. A large number of theoretical and experimental works have been devoted to the study of this effect [29–39].

By now, many such SSVs using various elemental metals and alloys have been studied in sufficient detail, and recent results indicate that significant values of the SSV effect have already been achieved in F1/F2/S structures [35–36,39]. Since the principle of a SSV relies on the S/F proximity effect, which is confined to the interface between the S and F layers, particular attention was paid to the quality of this interface in terms of its morphology, smoothness, and absence of intergrowth, which defines the mainstream approach in this field. At odds with this approach, a significant SSV effect of Δ*T*_c_ ≈ 0.3 K in an FeNi/In/Ni heterostructure with intentionally oxidized F/S interfaces was demonstrated by Deutscher and Meunier in 1969 [40]. The idea behind the oxidation of the FeNi and Ni layers was to slightly weaken the S/F proximity effect such that the superconductivity in the In layer could not be completely destroyed by the exchange field of the F layers. The authors noted that the thin oxidized layers became insulating but presumably remained magnetic. In a later experiment by Li et al. [41], the F layers themselves were insulating by design. In this special situation, even a very thin additional nonmagnetic insulating interlayer at the interface immediately suppressed the S/F proximity effect.

The paradoxical fact that “worsening” of the S/F interface in a metallic system [40] can yield a significant magnitude of the SSV effect is remarkable. The work by Deutscher and Meunier [40] has never been reproduced, and the research in this direction was not pursued; albeit, according to private communications in the SSV community, some groups attempted, but did not succeed, to reproduce this early remarkable result.

In the present work, in order to verify the SSV effect reported by Deutscher and Meunier for heterostructures in which the superconducting layer is contacted to the ferromagnets through thin insulating interlayers and to prove the validity of this concept for other types of SSV structures, we investigated the superconducting properties of a SSV made of F and S materials completely different from those in [40]. Specifically, we prepared Co1/Pb/Co2 multilayers with oxidized Co1/Pb and Pb/Co2 interfaces following the recipe of [40,42]. We studied the dependence of the magnitude of the SSV effect Δ*T*_c_ on the Pb layer thickness and found that Δ*T*_c_ reached 0.2 K for the optimal thickness, surpassing most of the values previously observed for SSVs with perfect metallic contact. We discuss the obtained results in the context of the existing theoretical models of the S/F proximity effect.

## Samples

For our investigation CoO*_x_* (3.5 nm)/Co1 (3 nm)/I1/Pb(*d*_Pb_)/I2/Co2 (3 nm)/Si_3_N_4_ (85 nm) heterostructures with variable Pb layer thickness *d*_Pb_ in the range from 60 to 120 nm were fabricated on high-quality single-crystalline MgO(001) substrates. Here, Co1 and Co2 are ferromagnetic F1 and F2 layers, I1 and I2 are thin oxide insulating interlayers, Pb is the superconducting layer, Si_3_N_4_ is a protective layer, and CoO*_x_* is the antiferromagnetic (AF) bias layer that fixes the direction of the magnetization of the Co1 layer. The layers were deposited using electron beam evaporation (Co, Pb) and AC sputtering (Si_3_N_4_). The deposition setup had a load-lock station with vacuum shutters, allowing one to transfer the sample holder without breaking the ultrahigh vacuum in the deposition chambers. The load-lock station provides the possibility to oxidize the prepared layers in a controlled atmosphere. This allows one to prepare the AF CoO*_x_* layer and to fabricate the thin oxide interlayers (I1 and I2) at the Co1/I1/Pb and Pb/I2/Co2 interfaces. CoO*_x_* was prepared by exposing the metallic Co layer to oxygen atmosphere at 100 mbar for two hours. Next, Co1 was deposited in the main deposition chamber at a vacuum pressure of the order of 10^−9^ mbar on top of the CoO*_x_* layer. The I1 layer was formed on the surface of Co1 in a similar way as described above in an oxygen atmosphere of ≈10^−2^ mbar for 60 s. It was shown in [43] that significant partial oxidation of a few nanometers thin metallic Co layer can be achieved by exposing it to the ambient atmospheric environment, implying that lowering the atmospheric pressure by five orders of magnitude enables one to oxidize only the surface without affecting the bulk of the layer. After that, the Pb layer and subsequent layers of the SSV structure were deposited at the substrate temperature of *T*_sub_ ≈ 150 K. Such low *T*_sub_ was necessary to obtain a smooth Pb layer [44] and to form the I2 layer. A similar oxidation procedure was used again to form the I2 layer by exposing the Pb surface to an oxygen atmosphere of ≈10^−2^ mbar for 30 s. After that, the Co2 layer was deposited similar to the Co1 layer.

According to [40,42] the O_2_ molecules adsorbed on the surface of the superconducting Pb layer oxidize the top ferromagnetic Co2 layer during its deposition, thereby, forming an insulating magnetic interlayer at the S/F interface. We consider an oxidation of the Pb layer to be unlikely because it was deposited at a low substrate temperature and exposed to a very low atmospheric pressure for a very short time, as specified above. According to the literature, the formation of an oxide on the surface of the Pb film requires significantly higher temperatures and pressures, and much longer exposition times [45–47].

**Figure 1:**
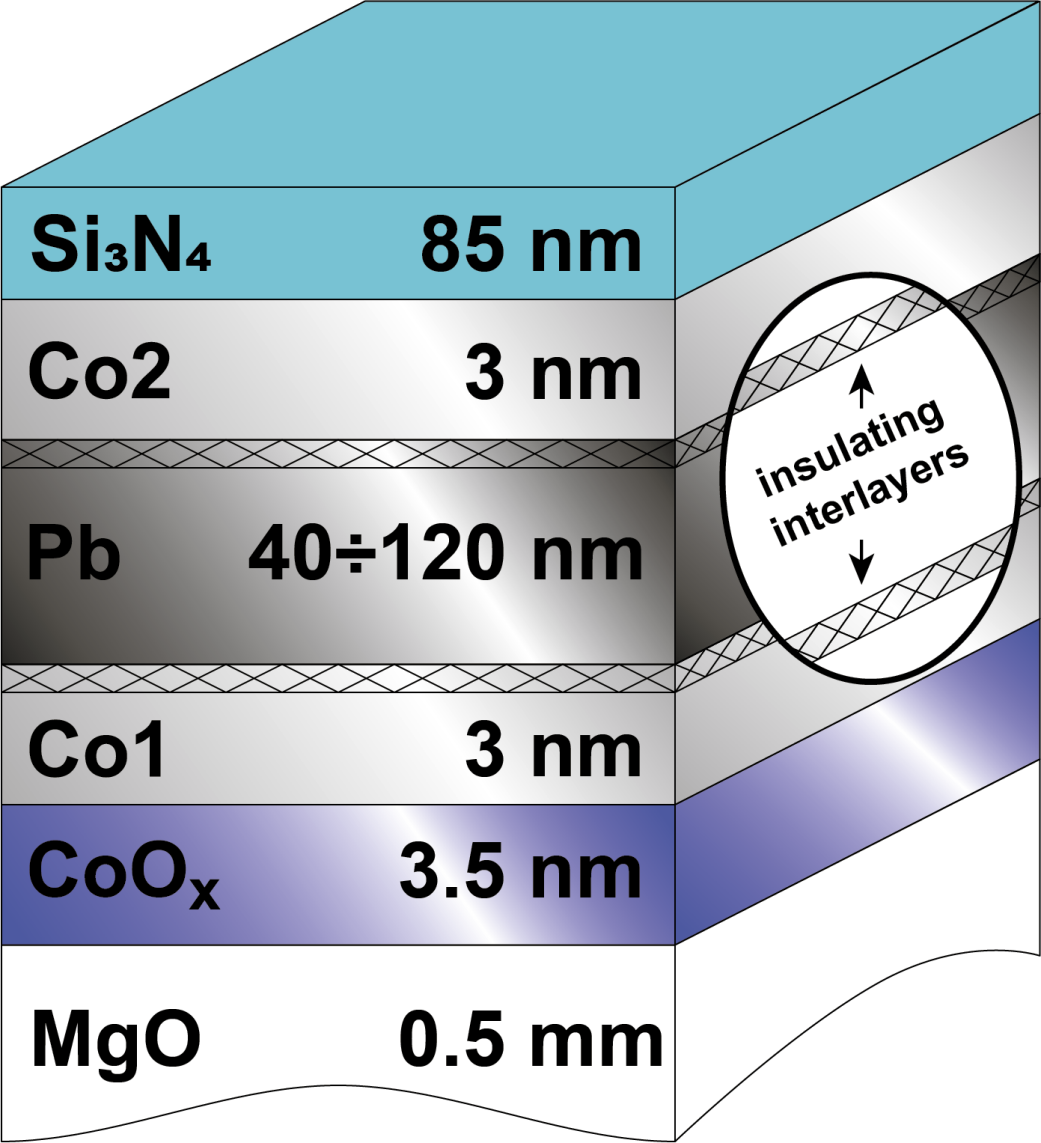
Design of the prepared samples. The cross-dashed areas depict the insulating interlayers I1 and I2 (see the text for details).

Finally, all samples were covered with a protective Si_3_N_4_ layer. The deposition rates were as follows: 0.5 Å/s for Co1 and Co2, 12 Å/s for Pb, and 1.8 Å/s for Si_3_N_4_ films. The final design of the samples is depicted in Figure 1.

Based on our previous studies in [33–34,48] we chose the thickness of the AF CoO*_x_* layer to be 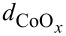 = 3.5 nm. This is optimal to maintain the direction of the magnetization of the Co1 layer up to an in-plane external magnetic field strength of 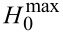 ≈ 1.5 kOe. Magnetic studies of the samples are presented in Supporting Information File 1. We took the same thickness of 3 nm for both Co1 and Co2 layers.

In addition, a control set of the samples with similar thicknesses of the S and F layers but without insulating interlayers at the Co1/Pb/Co2 interfaces was prepared for comparison. The list of the studied CoO*_x_* (3.5 nm)/Co1 (3 nm)/I1/Pb(*d*_Pb_)/I2/Co2 (3 nm)/Si_3_N_4_ (85 nm) samples with variable Pb layer thickness *d*_Pb_ is presented in Table 1.

**Table 1:** List of the studied samples CoO*_x_* (3.5 nm)/Co1 (3 nm)/I1/Pb(*d*_Pb_)/I2/Co2 (3 nm)/Si_3_N_4_ (85 nm) with the variable Pb layer thickness *d*_Pb_.

Samples with insulating interlayers	*d*_Pb_ (nm)

Pb_120	120
Pb_100	100
Pb_80	80
Pb_60	60
Pb_40	40

## Results

Electrical resistivity measurements were carried out with a standard four-point method in the DC mode. For changing the mutual direction of the magnetization of the F layers between the P and AP orientations, an external magnetic field of ≈1 kOe *<* 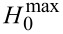 was always applied in the plane of the sample in all measurements. The strength of the magnetic field was measured by a Hall probe with an accuracy of ±0.3 Oe. The sample temperature was monitored using an Allen-Bradley thermometer that is highly sensitive in the temperature range of interest. The temperature measurement error was ±(5–6) mK below 3 K. The superconducting critical temperature *T*_c_ was defined as the midpoint of the transition curve.

To study the SSV effect, the samples were cooled down from room temperature to low temperatures in a magnetic field of the order of 5 kOe (field cooling procedure) applied in the sample plane. This field aligns the magnetization of both F layers. Also, the magnetization vector of the Co1 layer is getting fixed in the direction of the applied field and remains biased by the AF CoO*_x_* layer after the reduction of the field strength to its operational value of *H*_0_ = 1 kOe, independent of the subsequent direction of the field vector [33–34,48]. At this field value, the temperature dependence of the resistivity *R*(*T*) was recorded for the P and AP configurations of the magnetizations of the Co1 and Co2 layers by appropriate rotation of the magnetization of the Co2 layer through an external magnetic field.

**Figure 2:**
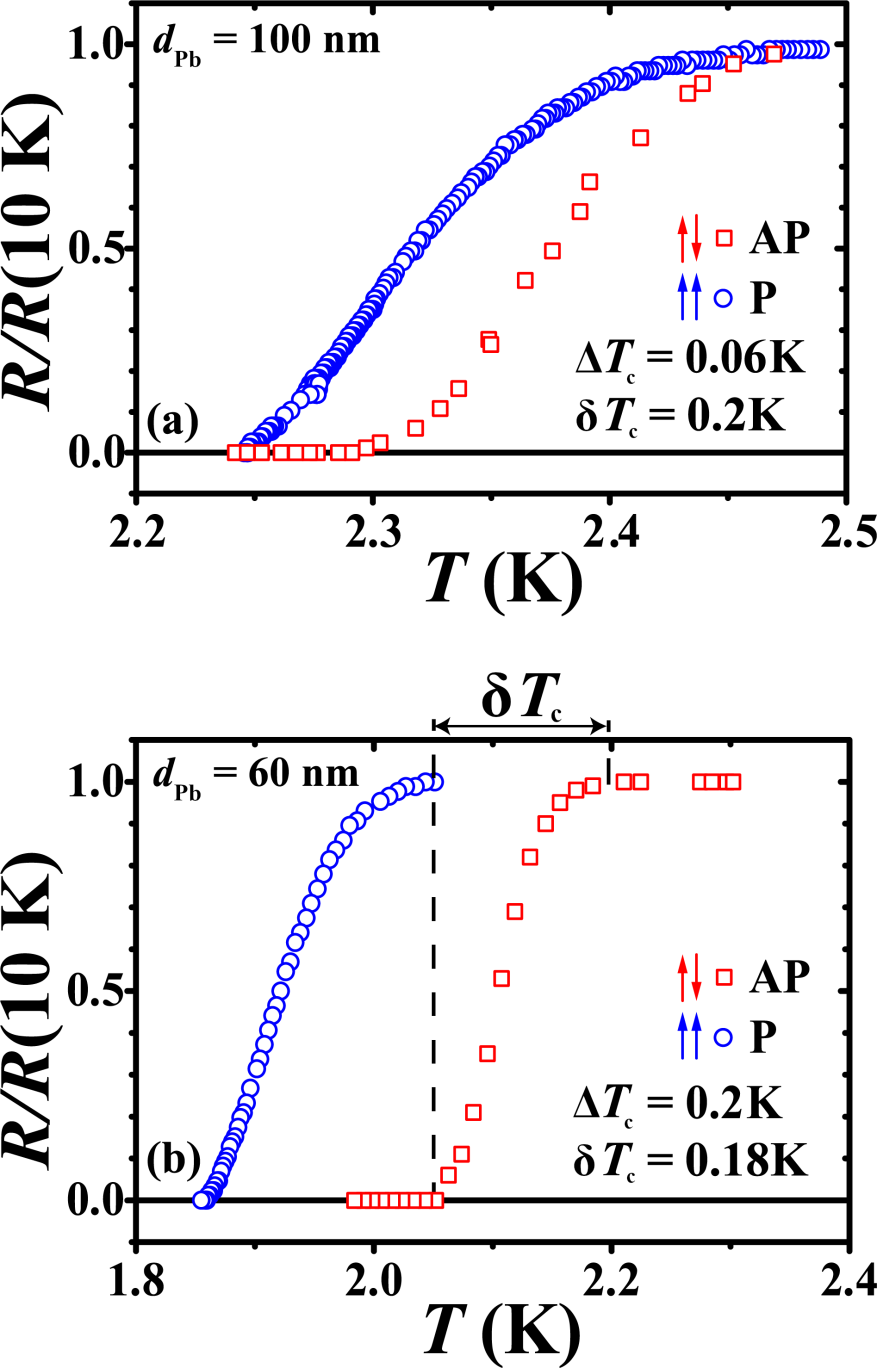
Superconducting transition curves for the samples Pb_100 (a) and Pb_60 (b) at P (*H*_0_ = +1 kOe) (circles) and AP (*H*_0_ = −1 kOe) (squares) orientations of the Co1 and Co2 layers's magnetizations, respectively.

Figure 2 depicts the superconducting transition curves for the samples Pb_100 and Pb_60 at P (*H*_0_ = +1 kOe) and AP (*H*_0_ = −1 kOe) orientations of the Co1 and Co2 layers’ magnetization, respectively. The magnitude of the SSV effect for the sample Pb_100 amounts to Δ*T*_c_ = 0.07 K, whereas for the sample Pb_60 , it rises up to Δ*T*_c_ = 0.2 K. Obviously, the sample Pb_60 demonstrates the full SSV effect since in this case Δ*T*_c_ *>* δ*T*_c_ as is evident from Figure 2b.

**Figure 3:**
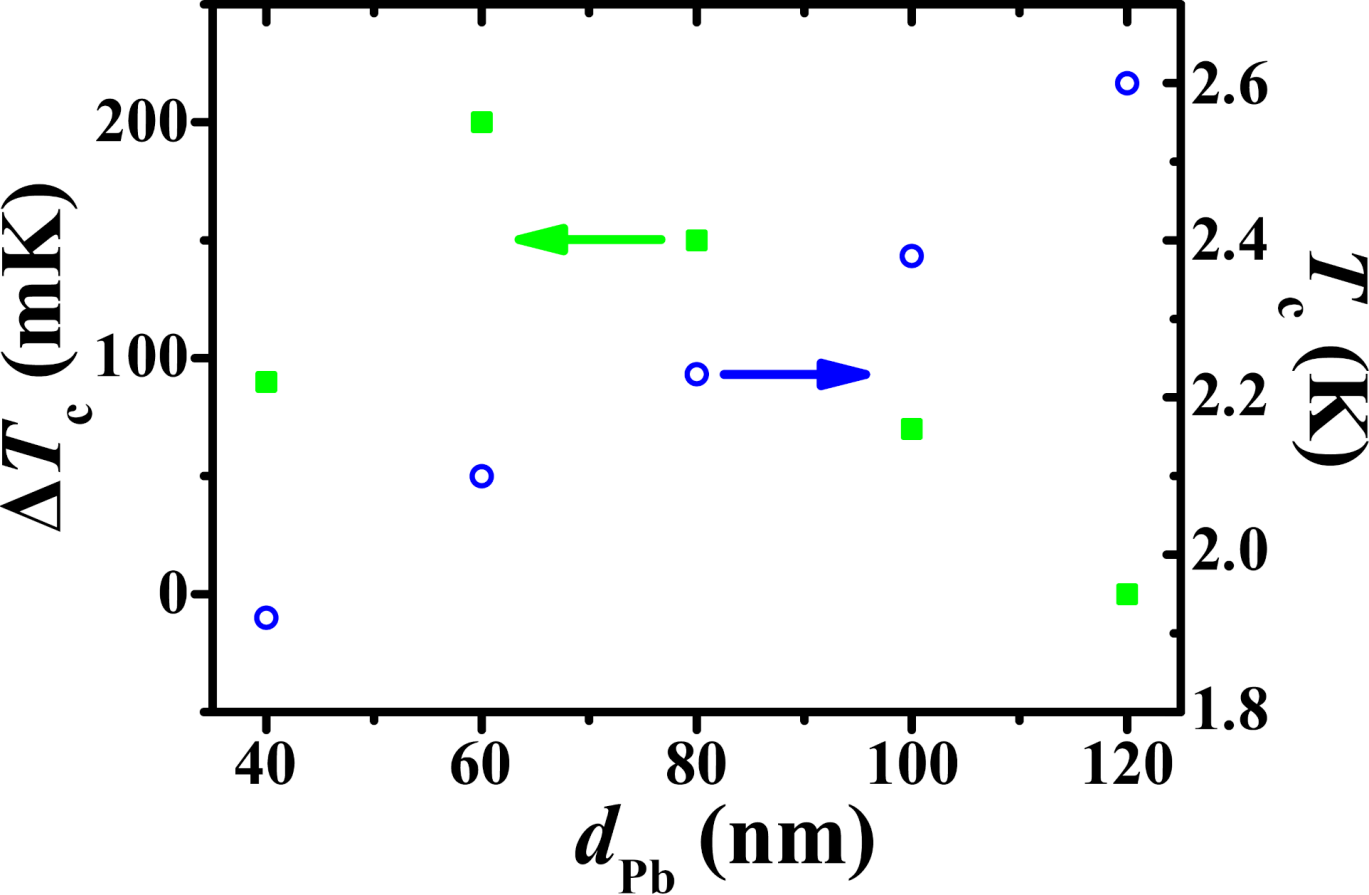
The dependence of the magnitude of the SSV effect Δ*T*_c_ (left vertical scale) and of the superconducting critical temperature 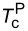 for the parallel orientation of the magnetizations of the Co1 and Co2 layers (right vertical scale) on the Pb layer thickness *d*_Pb_.

Figure 3 shows the dependence of Δ*T*_c_ and of *T*_c_ on the thickness of the Pb layer *d*_Pb_ for the whole set of samples with insulating interlayers. Δ*T*_c_ increases and *T*_c_ decreases approximately linearly with decreasing *d*_Pb_. The maximum magnitude of the SSV effect, Δ*T*_c_ = 0.2 K, is reached at the minimum thickness of the superconducting layer *d*_Pb_ = 60 nm.

Notably, in the control set of structures with similar parameters, but without the insulating interlayers, superconductivity was not observed down to the lowest temperature of the experimental setup of 1.4 K for all Pb thicknesses.

## Discussion

### Phenomenology

Our results demonstrate a significant SSV effect in heterostructures with insulating interlayers at the F1/S/F2 interfaces and finally verify the earlier observation by Deutscher and Meunier [40]. The S layer thickness appears to be an essential parameter for observing the full SSV effect. As *d*_Pb_ decreases, the value of Δ*T*_c_ increases and reaches its maximum of 0.2 K at *d*_Pb_ = 60 nm (Figure 3). The decrease of *T*_c_ as a function of *d*_Pb_ is approximately linear down to *d*_Pb_ = 40 nm. A sharp drop of *T*_c_ is expected at smaller thicknesses of the S layer due to the size effects [49]. Apparently, the inverse S/F proximity effect becomes more pronounced as *d*_Pb_ decreases, despite the existence of insulating interlayers. The here obtained value of Δ*T*_c_ at the optimal thickness of the Pb layer is twice as high compared to those found before in [30,33–34,48] for structures with elemental metallic ferromagnetic layers but without insulating interlayers.

This observation is not trivial as it apparently contradicts the paramount prerequisite of the S/F proximity effect of having a perfect metallic contact between the S and F layers. It is plausible that oxide insulating interlayers remain magnetic as suggested in [40,42]. They may play a dual role of attenuating the influence of the metallic ferromagnetic layer on the S layer, which completely suppresses superconductivity in our control fully metallic Co1/Pb/Co2 stacks, and at the same time maintaining some kind of the proximity effect that enables switching between the normal and superconducting states.

At the same time, the nontrivial S/F proximity effect in our system originates from metallic F layers. This is evidenced by the fact that similar control structures with reduced thickness of the Co layers (2 nm instead of 3 nm) did not show any spin valve effect.

Note that the on/off switching of superconductivity in the trilayer EuS/Al/EuS, where EuS is a ferromagnetic insulator, has been demonstrated by Li and coworkers [41]. This type of system is different from metallic-type structures of [40] and the present work, in which only very thin oxidized interfaces are insulating.

**Figure 4:**
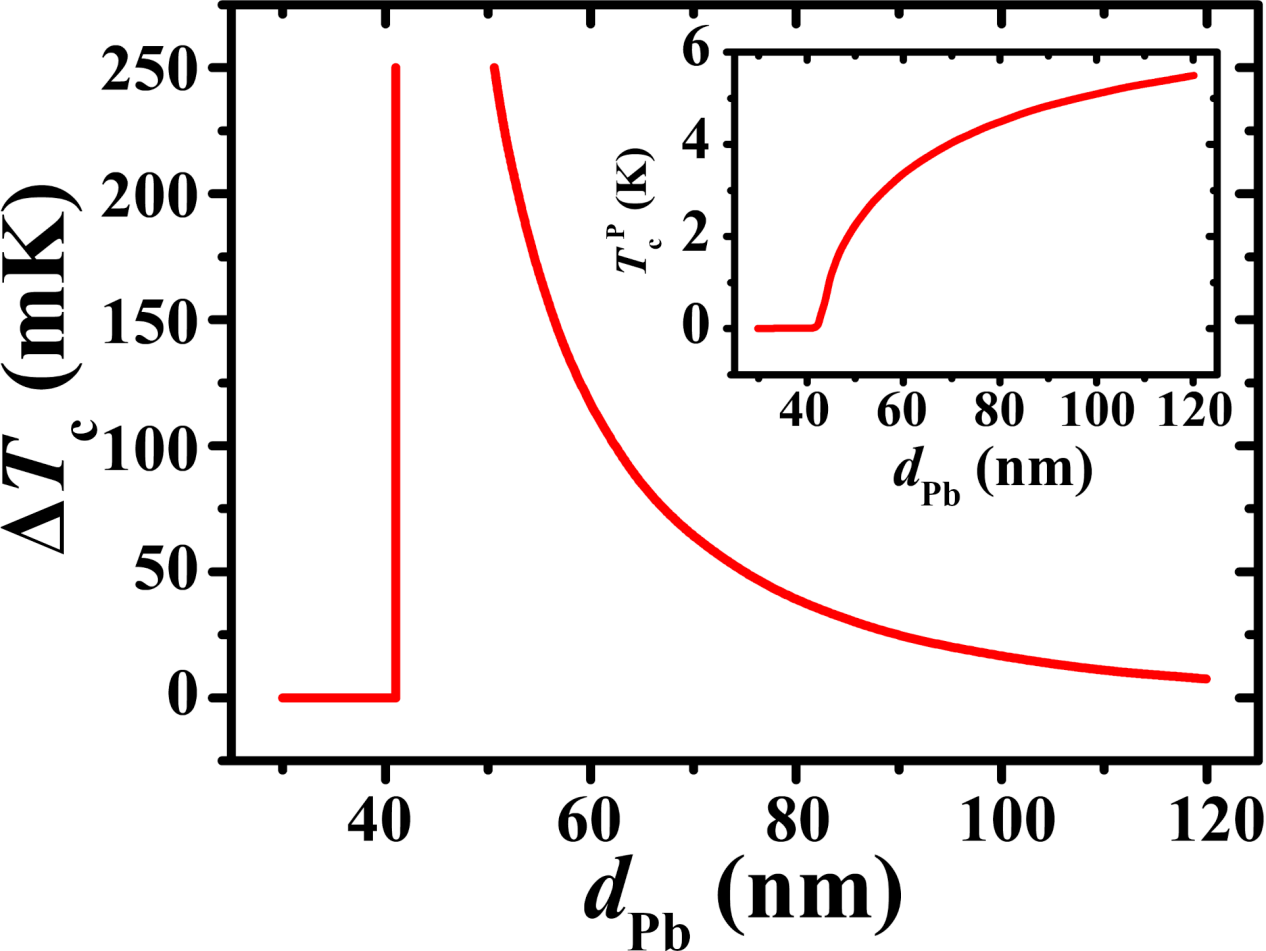
Modeling results for the Δ*T*_c_(*d*_Pb_) and 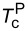
(*d*_Pb_) dependences in main figure and inset, respectively. The parameters of the model are as follows: ξ_S_ = 41 nm, ξ_F_ = 12 nm, *h* = 0.035 eV, γ = 0.093, and γ*_b_* = 0.48 (see the text and [50] for the exact definitions).

### Theoretical analysis

The modeling of the observed significant SSV effect in heterostructures with intentionally deteriorated S/F interfaces is rather challenging because of an increased complexity of these interfaces as compared with the ideal metallic contacts between the layers. Though experimentally the formation of an insulating interlayer by oxidation appears to be a doable task, their characteristics, such as thickness, exact composition, and physical properties, cannot be sufficiently well controlled at present. Therefore, in the following we will discuss whether the main tendencies of the SSV effect with regard to the thickness of the superconducting Pb layer (Figure 3) could be at least qualitatively captured by theory.

The proximity effect theory suitable for the description of *T*_c_ in symmetric F1/S/F2 structures was formulated in [50]. Applying this theory to our experimental data, under the abovementioned somewhat ambiguous conditions we can still achieve qualitative agreement with the experiment. In the case of the Δ*T*_c_(*d*_Pb_) dependence, see Figure 4, the theory demonstrates the nonmonotonicity of the dependence and the approximate position of the maximum. This maximum is expected since the spin valve effect should be suppressed both in the limit of very thin and very thick S layers, with a maximal value at a thickness *d*_S_ of the order of the coherence length ξ_S_. At the same time, the quantitative agreement between theory and experiment is not good, as expected. The same is true in the case of the 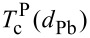 dependence plotted in the inset to Figure 4. The theoretical model predicts a sharp decline in *T*_c_ at a *d*_Pb_ value close to 40 nm, but the measurements suggest a smoother dependence of the critical temperature, possibly extending to lower temperatures. The model also suggests an asymmetric peak in Δ*T*_c_(*d*_Pb_), whereas a more symmetric peak is observed in Figure 3 (at the same time, the left side of the peak is steeper than the right one both in theory and in experiment). Finally, the model indicates a rapid convex-type decay of Δ*T*_c_(*d*_Pb_) to the right of the peak, whereas the experimental data suggests a linear dependence. At the same time, because of the limited number of experimental points, we cannot exclude a nonlinear behavior around the peak in Δ*T*_c_(*d*_Pb_) (in order to check this, points in the thickness range between 40 and 60 nm would be required).

The fitting parameters given in the caption to Figure 4 were obtained as follows. The coherence lengths in the S and F materials, ξ_S_ and ξ_F_, respectively, were estimated from the residual resistivities of the materials. The values of the exchange energy *h*, the materials-matching interface parameter, γ, and the interface resistance parameter, γ*_b_* were chosen in order to provide the correct position of the Δ*T*_c_ maximum and acceptable overall values of this quantity (cf. Figure 3). The same values were then employed to plot the theoretical curve for 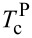
(*d*_Pb_). The value of γ is consistent with the values of ξ_S_ and ξ_F_.

What is unexpected in the above fitting parameters is a rather small value of the interface resistance parameter, γ_b_ = 0.48. In the tunneling limit, one can estimate 
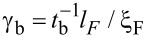
 in terms of the effective interface transparency *t*_b_ ≪ 1 (while *l*_F_ is the mean free path in the F material). The transparency values are not directly measurable. At the same time, it is known that in the case of conventional tunnel junctions with insulating interfaces of thickness from 10 to 30 atomic layers, the order of magnitude of *t*_b_ varies between 10^−3^ and 10^−5^ [51]. In that case, we would expect larger values of γ_b_ than the one resulting from our fit. However, we have checked that larger values of γ_b_ notably suppress the sensitivity of *T*_c_ to the presence of the F layers (i.e., the inverse proximity effect becomes strongly suppressed). The obtained small value of γ_b_ points at small thicknesses of the tunneling barriers in our junctions. Note that this correlates with observations by Deutscher and Meunier [40], who concluded that according to the resistance measurements, the barriers in their experiment were “much thinner than in a conventional tunneling junction”.

While the theory [50] assumes a symmetric F1/S/F2 structure, our samples may actually be asymmetric from the point of view of the interface transparencies. The oxidation times of the two interfaces were different in our samples, and our fabrication procedure was such that the oxidation affected different materials (first, Co1 was oxidized, then Pb) at different temperatures. However, a generalization of our theory to the case of two different γ_b_ parameters is still expected to suppress the proximity effect almost completely in the case of two tunneling interfaces (while already one tunneling interface with *t*_b_ ≪ 1 should effectively “detach” the corresponding F layer and, thus, suppress the effect of rotating magnetization on *T*_c_).

A possible reason for not too small transparencies following from the fit is that the insulating layers in our samples are actually very thin (a few atomic layers). Another possibility is that the resulting oxides are not good insulators but possess finite conductivity or that metallic shortcuts are present inside the insulating layers. Finally, in contrast to our theory [50] assuming nonmagnetic insulating barriers, the interfaces could be magnetically active [52], which would introduce additional degrees of freedom into the system (in particular, the interfaces could then behave nontrivially under the action of a rotating magnetic field). Further experiments with better control of the insulating interfaces are clearly needed in order to clarify the role of the oxidized interfaces.

## Conclusion

In summary, we have investigated superconducting properties of Co1/Pb/Co2 SSV heterostructures with thin insulating oxide interlayers formed at the Co1/Pb and Pb/Co2 interfaces. We found the optimal thickness of the superconducting Pb layer for the realization of the full superconducting spin valve effect with a magnitude of Δ*T*_c_ = 0.2 K. Our finding finally substantiates the results of the earlier work by Deutscher and Meunier [40], where a surprisingly large SSV effect was found for F1/S/F2 structures with insulating interlayers. It is remarkable that the here obtained value of Δ*T*_c_ significantly exceeds those of many of the multilayers prepared of elemental metallic ferromagnets and superconductors, where special care was taken to achieve a perfect metallic contact at the S/F interface in order to enhance the S/F proximity effect.

Also, for the spin valve effect, the key parameter is not the strength of the proximity effect, but rather the sensitivity of the system to the variation of the relative magnetizations. Our strategy was to achieve a “fragile” superconductivity, which is sensitive to this kind of control. To this end, we have realized systems with such parameters that superconductivity is completely suppressed in the limit of perfect metallic interfaces. The role of the insulating interface layers is then to restore superconductivity in the system. This fragile “restored” superconductivity turns out to be indeed very sensitive to the configuration of the F part of the structure.

Our findings thus call for further exploration of this promising route to improve the operational parameters of the superconducting spin valves by advancing the preparation technologies and developing the underlying theories.

## Supporting Information

File 1 Characteristic magnetic hysteresis loops *M*(*H*) for the samples with and without insulating interlayers.

## Data Availability

All data that supports the findings of this study is available in the published article and/or the supporting information to this article.
