# Supplementary material for: Superconducting spin valve effect in Co/Pb/Co heterostructures with insulating interlayers
Source: Beilstein J Nanotechnol. 2024 Apr 25;15:457–64. doi: 10.3762/bjnano.15.41 (PMC11070957; doi:10.3762/bjnano.15.41)
Supplement: File 1 — Characteristic magnetic hysteresis loops M(H) for the samples with and without insulating interlayers. [file Beilstein_J_Nanotechnol-15-457-s001.pdf]

BEILSTEIN JOURNAL OF NANOTECHNOLOGY

Supporting Information

for

Superconducting spin valve effect in Co/Pb/Co heterostructures with insulating interlayers

Andrey A. Kamashev, Nadir N. Garif'yanov, Aidar A. Validov, Vladislav Kataev, Alexander S. Osin, Yakov V. Fominov and Ilgiz A. Garifullin

Beilstein J. Nanotechnol. **2024,** *15,* 457–464. doi:10.3762/bjnano.15.41

Characteristic magnetic hysteresis loops *M*(*H*) for the samples with and without insulating interlayers

License and Terms: This is a supporting information file under the terms of the Creative Commons Attribution License (https://creativecommons.org/licenses/by/4.0). Please note that the reuse, redistribution and reproduction in particular requires that the author(s) and source are credited and that individual graphics may be subject to special legal provisions.
The license is subject to the *Beilstein Journal of Nanotechnology* terms and conditions: (https://www.beilstein-journals.org/bjnano/terms)

Figure S1 presents characteristic hysteresis $M(H)$ loops for the samples with and without insulating interlayers. The curves appear very similar in particular demonstrating three distinct geometries of the magnetization vectors of the F1 and F2 layers as illustrated in this Figure. The samples were cooled down in a positive field of ~ 5 kOe which was then swept down towards negative values. The freely rotating magnetization of the F2 layer is immediately flipped by crossing $H = 0$ yielding a step in the $M(H)$ curve. The magnetization of the F1 layer remains pinned in the positive direction and is flipped in a much stronger negative field yielding the second step in the $M(H)$ curve. This flipping field is reduced from ~ -6 kOe in the control sample to ~ -2 kOe in the SSV sample presumably due to the partial oxidation of the Co1 layer. However, at the operational fields ± 1 kOe used to demonstrate the SSV effect in the samples with oxidized interlayers the magnetization of the Co1 layer remains biased which enables switching between parallel and antiparallel orientation of the magnetization vectors of the F1 and F2 layers.

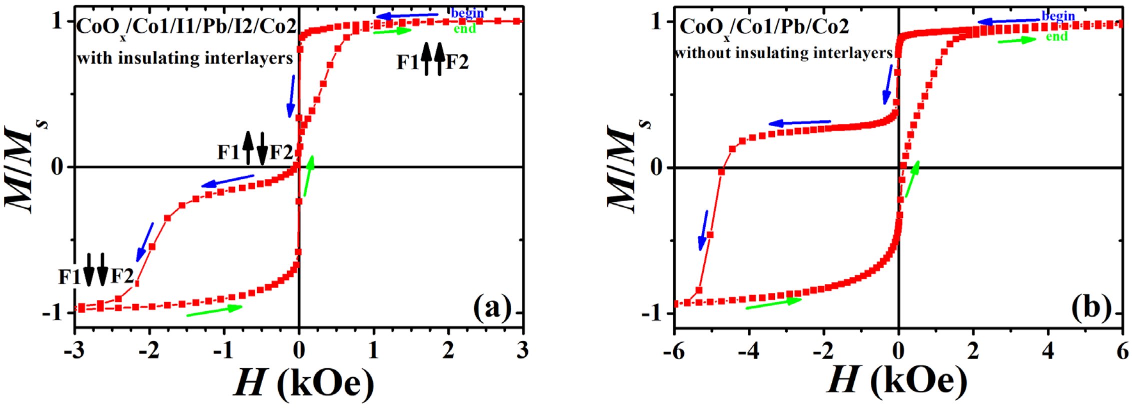

Figure S1: Characteristic hysteresis loops for CoO_x/Co1/I1/Pb/I2/Co2 (SSV with insulating interlayers) (a) and CoO_xCo1/Pb/Co2 (SSV without insulating interlayers) (b) types of samples.
